# Successful prevention of balloon dilatation after complete circumferential endoscopic submucosal dissection including long-segment Barrett’s esophagus

**DOI:** 10.1055/a-2346-4577

**Published:** 2024-07-03

**Authors:** Kenichiro Okimoto, Tomoaki Matsumura, Keisuke Matsusaka, Yuki Ohta, Takashi Taida, Jun Kato, Naoya Kato

**Affiliations:** 1 Department of Gastroenterology, Graduate School of Medicine, Chiba University, Chiba, Japan; 2 12737 Department of Pathology, Chiba University, Chiba, Japan; 3 92154 Endoscopy Center, Chiba University Hospital, Chiba, China

Triamcinolone acetonide (TA) injection into submucosa is useful for prevention of stricture after esophageal endoscopic submucosal dissection (ESD) 11 . However, despite attempts with steroid use, complete circumferential esophageal ESD still leads to high stricture rates (36.4% to 85.7% 22 33 44 ). Although endoscopic balloon dilatation (EBD) is often necessary for stricture relief, it carries the risk of perforation 22 . In addition, in long-segment Barrett’s esophagus (BE) with Barrett's esophageal adenocarcinoma (BEA), post-ESD ulcers can be too long, making EBD challenging. Thus, alternative stricture methods preventing EBD are needed.

Here we present a case of successful prevention of EBD after a circumferential ESD for BEA in long-segment BE (longitudinal length of resected area was 12 cm endoscopically) with intensive TA injections ( Video 1Video 1 ). ESD utilized MucoUp (Seikagaku, Tokyo, Japan and Boston Scientific Japan, Kanagawa, Japan) with indigo carmine, 1.5-mm DualKnife J (Olympus Medical Systems, Tokyo, Japan), and the clip-and-line method 55 . En bloc resection, including the entire long-segment BE (Prague classification C8M9), was performed ( Fig. 1Fig. 1 ). The pathological finding was BEA with unclear horizontal margin and negative vertical margin ( Fig. 2Fig. 2 ).

Weekly intensive triamcinolone acetonide injections were beneficial for preventing stricture in complete circumferential esophageal endoscopic submucosal dissection.Video 1Video 1

**Fig. 1:**
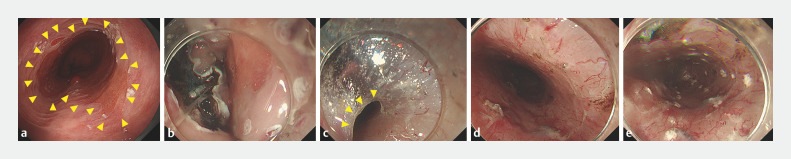
**Fig. 1** The details of endoscopic submucosal dissection (ESD). **a** The patient was diagnosed with circumferential Barrett's adenocarcinoma with long-segment Barrett’s esophagus (BE) (C8M9 according to the Prague classification). The yellow arrows indicate the proximal boundary of long-segment BE. **b** ESD was performed with the clip-and-line method. **c** ESD was performed by creating a submucosal tunnel. The yellow arrows indicate the edge of the submucosal tunnel. **d** Immediately after ESD, en bloc resection was performed including the entire long-segment BE. The longitudinal length of the post-ESD defect measured endoscopically was up to 12 cm from the oral to the anal side. **e** A total of 100 mg of triamcinolone acetonide (TA) was locally injected into the remaining submucosa.

**Fig. 2:**
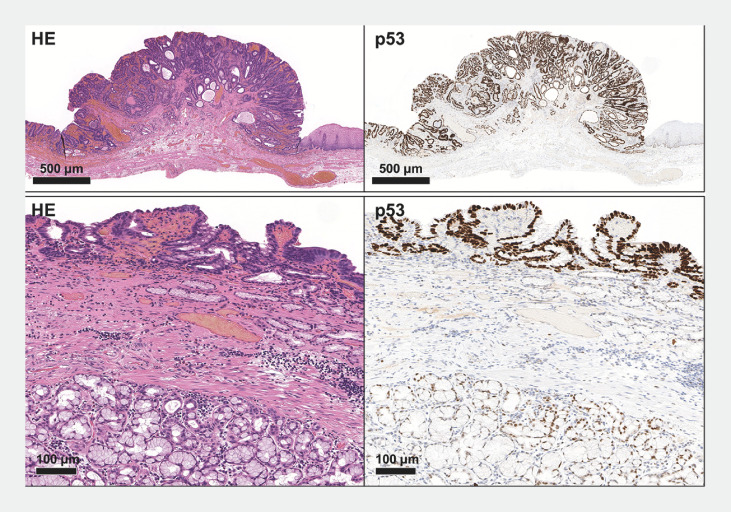
**Fig. 2** Pathological findings of the resected specimen. Histological photograph of an adenocarcinoma at the esophagogastric junction. Atypical glandular epithelium forms irregular tubules, creating polypoid, protruding lesions. Immunostaining shows a mutant pattern of p53 overaccumulation. Meanwhile, a flatly spreading adenocarcinoma is observed around the protrusions. Submucosal esophageal glands in the columnar epithelium region are identified, suggesting an adenocarcinoma arising in Barrett's esophagus.

TA (KENACOLT-A 50 mg/5 mL; Bristol Myers Squibb, Tokyo, Japan) was diluted to 5 mg/mL with normal saline. A 26-gauge 4-mm needle (SG-A 26G FE 4 mm 2200 mm; TOP Corporation, Tokyo, Japan) was used for injection. TA injection into the submucosa, starting immediately post-ESD, was performed at intervals of 0.5 mL (TA 2.5 mg), preventing injury to the muscularis propria. Subsequent injections occurred 3 days post-ESD and then weekly for 21 weeks, with additional injections on weeks 23 and 25, totaling 25 sessions. A total of 50–100 mg TA was administered in each session. At 40 weeks post-ESD, complete epithelialization without stricture was achieved ( Fig. 3Fig. 3 ).

**Fig. 3:**
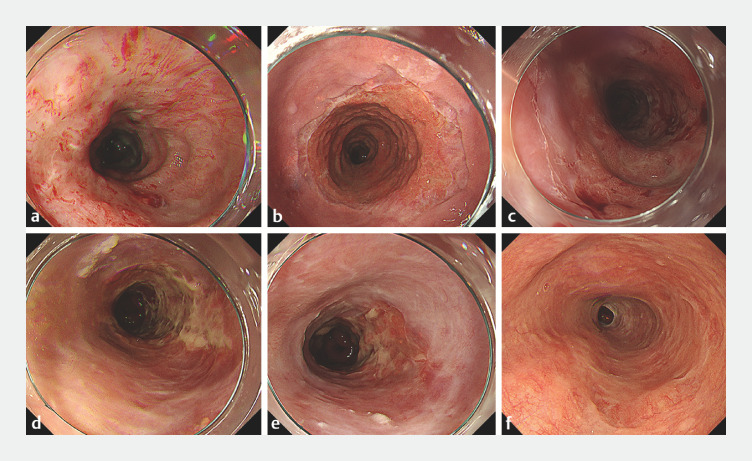
**Fig. 3** The healing process of the post-ESD ulcer. The epithelialization progressed in chronological order from Fig. 3Fig. 3 **a–f** . A total of 50 mg or 100 mg of TA was locally injected into the regenerating submucosal layer from Fig. 3Fig. 3 **a–e** , respectively. **a** 1 week after ESD (3rd local injection of TA). **b** 4 weeks after ESD (6th local injection of TA). **c** 8 weeks after ESD (10th local injection of TA). **d** 12 weeks after ESD (14th local injection of TA). **e** 20 weeks after ESD (22nd local injection of TA). **f** 40 weeks after ESD. Complete epithelialization without any stricture was achieved, and a φ9.8-mm scope (GIF-H290T; Olympus Medical Systems, Tokyo, Japan) passed easily.

Weekly intensive TA injections alone were beneficial for preventing stricture in complete circumferential esophageal ESD even if the resected area was long.

Endoscopy_UCTN_Code_TTT_1AO_2AO

## Notes

Endoscopy E-Videos https://eref.thieme.de/e-videos
*E-Videos* is an open access online section of the journal *Endoscopy* , reporting on interesting cases and new techniques in gastroenterological endoscopy. All papers include a high-quality video and are published with a Creative Commons CC-BY license. Endoscopy E-Videos qualify for HINARI discounts and waivers and eligibility is automatically checked during the submission process. We grant 100% waivers to articles whose corresponding authors are based in Group A countries and 50% waivers to those who are based in Group B countries as classified by Research4Life (see: https://www.research4life.org/access/eligibility/ ). This section has its own submission website at https://mc.manuscriptcentral.com/e-videos .
